# Inferring haplotypes at the *NAT2 *locus: the computational approach

**DOI:** 10.1186/1471-2156-6-30

**Published:** 2005-06-02

**Authors:** Audrey Sabbagh, Pierre Darlu

**Affiliations:** 1 Unité de Recherche en Génétique Epidémiologique et Structure des Populations Humaines, INSERM U535, Villejuif, France

## Abstract

**Background:**

Numerous studies have attempted to relate genetic polymorphisms within the N-acetyltransferase 2 gene (*NAT2*) to interindividual differences in response to drugs or in disease susceptibility. However, genotyping of individuals single-nucleotide polymorphisms (SNPs) alone may not always provide enough information to reach these goals. It is important to link SNPs in terms of haplotypes which carry more information about the genotype-phenotype relationship. Special analytical techniques have been designed to unequivocally determine the allocation of mutations to either DNA strand. However, molecular haplotyping methods are labour-intensive and expensive and do not appear to be good candidates for routine clinical applications. A cheap and relatively straightforward alternative is the use of computational algorithms. The objective of this study was to assess the performance of the computational approach in *NAT2 *haplotype reconstruction from phase-unknown genotype data, for population samples of various ethnic origin.

**Results:**

We empirically evaluated the effectiveness of four haplotyping algorithms in predicting haplotype phases at *NAT2*, by comparing the results with those directly obtained through molecular haplotyping. All computational methods provided remarkably accurate and reliable estimates for *NAT2 *haplotype frequencies and individual haplotype phases. The Bayesian algorithm implemented in the PHASE program performed the best.

**Conclusion:**

This investigation provides a solid basis for the confident and rational use of computational methods which appear to be a good alternative to infer haplotype phases in the particular case of the *NAT2 *gene, where there is near complete linkage disequilibrium between polymorphic markers.

## Background

N-acetylation polymorphism is one of the earliest discovered and most intensively studied pharmacogenetic traits that underlie interindividual and interethnic differences in response to xenobiotics. In humans, acetylation is a major route of biotransformation for many arylamine and hydrazine drugs, as well as for a number of toxins and known carcinogens present in the diet, cigarette smoke and the environment [1-3]. Genetically determined differences in N-acetylation capacity have been proved to be important determinants of both the effectiveness of therapeutic response and the development of adverse drug reactions and toxicity during drug treatment [4]. In the last decades, numerous investigations have been made to elucidate the genetic basis of N-acetylation polymorphism in various ethnic groups in order to develop efficient genotyping tests and to adapt therapies to specific patients and populations in accordance with their genetic makeup. Some of the drugs excreted by acetylation are indeed crucial in the treatment of diseases representing a worldwide concern, such as tuberculosis and AIDS-related complex diseases [5,6]. Moreover, a number of epidemiological studies have suggested possible associations between the N-acetylator phenotype and a variety of complex human diseases, the most consistent findings being those regarding urinary bladder cancer and familial Parkinson's disease [7-10].

The gene coding for the arylamine N-acetyltransferase 2 (*NAT2*) enzyme has been established as the site of the classic human acetylation polymorphism [11-13] and the molecular basis of individual and interethnic variation in acetylation capacity is now well documented [14,15]. All mutations reported to date are found within the 870-bp coding region of the *NAT2 *gene. Among the seven single nucleotide polymorphisms (SNPs) that are commonly found in human populations, four result in an amino acid substitution that leads to a significant decrease in acetylation capacity (single base-pair substitutions at positions 191, 341, 590, 857). The other three are either silent mutations (C282T, C481T) or a non-synonymous substitution that does not alter phenotype (A803G).

In the consensus gene nomenclature of human *NAT2 *that encompasses all currently recognized alleles [16,17], sets of SNPs located throughout the coding region are linked in terms of haplotypes, that is they are organized as they segregate together on one individual's chromosome at the *NAT2 *locus. Each combination of SNPs identified so far constitutes a distinct haplotype that is treated as an allele of the haplotype system. The consideration of multilocus haplotypes seems more desirable since there is growing evidence that for genes containing multiple SNPs in high linkage disequilibrium (LD) such as *NAT2 *[18], haplotype structure rather than individual SNPs can be the principal determinant of phenotypic consequences [19-21]. A functional polypeptide is indeed the product of a haplotype, covering the entire coding region and coded by a single chromosome.

The *NAT2 *alleles described so far contain up to four of the acknowledged mutations in various combinations. Each allele is associated with an acetylator phenotype depending on which mutations they contain: for instance, substitutions at positions 191, 341, 590, and 857 are diagnostic for defective *NAT2 *function and hence for the slow acetylator phenotype (Table 1). Three *NAT2 *phenotypes have been described: subjects with two low activity alleles are classified as slow acetylators, while those with two functional alleles are considered rapid acetylators. If only one allele is of the slow type, an intermediate phenotype is observed. Many early studies did not distinguish between fast and intermediate acetylators, categorizing both types of subjects as fast acetylators.

**Table 1:** The major human *NAT2 *alleles and their associated phenotype^a^.

Allele	Nucleotide change ^b^							Phenotype
	**G191A**	C282T	**T341C**	C481T	**G590A**	A803G	**G857A**	
NAT2*4								rapid
NAT2*5A			**x**	x				slow
NAT2*5B			**x**	x		x		slow
NAT2*5C			**x**			x		slow
NAT2*6A		x			**x**			slow
NAT2*6B					**x**			slow
NAT2*7A							**x**	slow
NAT2*7B		x					**x**	slow
NAT2*12A						x		rapid
NAT2*12B		x				x		rapid
NAT2*13		x						rapid
NAT2*14A	**x**							slow
NAT2*14B	**x**	x						slow

Nucleotide substitutions shown in bold have a functional consequence on enzyme activity. Bold-faced alleles contain functional polymorphisms and are hence associated with the slow acetylator phenotype. Classification of *NAT2 *alleles into different clusters is based on the most functionally significant nucleotide substitution present: the NAT2*5, NAT2*6, NAT2*7 and NAT2*14 clusters possess signature nucleotide substitutions at positions 341, 590, 857 and 191, respectively and are hence all decreased function alleles ('slow alleles'). The others display enzymatic activity comparable to the rapid acetylator allele NAT2*4. There are significant interethnic differences in *NAT2 *allele distribution and frequency [14, 15].

^a ^Adapted from Hein et al. [3]. *NAT2 *nomenclature is accessible on the internet at website

^b ^Only changes from the reference sequence (NAT2*4) are indicated.

**Figure 1:**
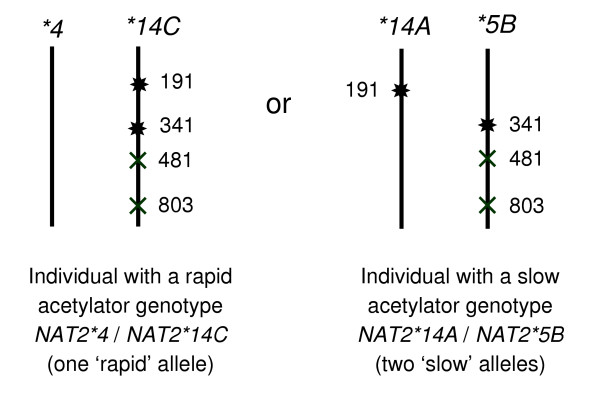
The ambiguous gametic phase of haplotypes for a given multilocus genotype. To illustrate the relevance of linkage phase ascertainment, let us consider the following case of a four-site heterozygous individual at positions 191, 341, 481 and 803 within the *NAT2 *coding sequence. Eight possible combinations of haplotypes can be inferred from this multilocus genotype, two of whom are shown here. Depending on the location of mutations to either DNA strand, the individual's *NAT2 *genotype composed of two multilocus haplotypes will not be the same. Moreover, an incorrect resolution of mutation linkage patterns may entail an error in individual phenotype prediction: the subject will be classified either as a slow or as a rapid acetylator depending on the haplotypic combination chosen. Symbol (*) points at mutations leading to a decrease in *NAT2 *enzyme activity, while symbol (**×**) indicates those with no impact on the acetylator phenotype.

Problems may occur when individual multi-site *NAT2 *genotypes have to be assigned correctly to a particular combination of two multilocus haplotypes. Indeed, current routine genotyping and sequencing methods typically do not provide haplotype information in diploid organisms such as humans, and the gametic phase of haplotypes is inherently ambiguous when individuals are heterozygous at more than one locus. As illustrated in Figure 1, a subject carrying two inactivating mutations can be either rapid or slow acetylator depending on whether these mutations are located in the same or different chromosome, respectively. It is thus crucial to unequivocally assess mutation linkage patterns, this step being a prerequisite to obtain accurate haplotype frequency estimates in populations and reliable genotype-phenotype predictions.

However, in spite of its high relevance, this issue has not been handled properly by most past studies investigating *NAT2 *polymorphisms. Early genotyping studies only screened for the presence of three polymorphisms (C481T, G590A, G857A), and a subject was defined as a slow acetylator if he was homozygous for one, or heterozygous for two (each located on one DNA strand), of this three nucleotide changes. Such a definition assumed that there could be no single allele with two or more of the tested mutations. In other studies that screened a larger number of SNPs within *NAT2*, patterns of LD between point mutations were often assumed, in reference to the haplotypes previously described and which are commonly found in populations of European origin. For instance, the designation of *NAT2 *alleles is usually based on the assumption that 481T and 803G are strongly linked to 341T, and 590A and 857A are linked to 282T [22]. However, in rare cases the typical allelic linkage pattern of mutations may be disrupted because of genetic recombination and this may result in misclassification of alleles. Indeed, although such assumed linkage patterns are very strong, other allelic variants carrying either unusual combinations of mutations or mutations in isolation have been described in a few cases [23]. Furthermore, the designation of *NAT2 *alleles in such a way that it necessarily conforms to the existing consensus nomenclature of acknowledged haplotypes precludes the disclosure of unexpected combinations of mutations, different from the established allelic variants, and hence, the discovery of new alleles. Such a manner of inferring haplotypes from unphased multilocus genotypes may introduce biases in *NAT2 *allele designation and individual phenotype prediction, and these potential biases are of higher magnitude when non-European populations are concerned. Most studies assumed particular patterns of linkage previously described in populations of European origin, but which may not hold in other ethnic groups. Indeed, recent works have shown that patterns of LD can differ markedly among populations with different ethnic and demographic backgrounds. As an example, Loktionov and colleagues [24] pointed out the high occurence of isolated mutations 803G and 282T (defining alleles NAT2*12A and NAT2*13, respectively) in Black South Africans, while these nucleotide changes are almost always tightly linked to other mutations in European populations [25]. Likewise, Dandara *et al. *[26] recently identified a novel mutation linkage pattern (NAT2*6E) that appeared to be common in three African populations and that had not yet been reported in Europeans. As well, Anitha *et al. *[27] revealed a new combination of acknowledged mutations (NAT2*5G) in the Malapandaram tribe of South India that has not been described so far in any other world population. The genotype-phenotype discordance observed in many ethnic groups where mutation linkages have not been extensively proven experimentally might result from such unexpected compound alleles. It is thus necessary to systematically verify the postulated allelic combinations.

To avoid such potential problems, many authors designed special analytical techniques to unequivocally determine the allocation of mutations to either DNA strand. Molecular methods using combinations of mutation-specific polymerase chain reaction (PCR) reamplification coupled to restriction mapping of the PCR products have been developed; these allow the separate analysis of each allele in order to obtain a complete map of both genes in every individual. Some studies applied these procedures to all multiply heterozygous subjects [24,28-31], while others limited their application to particular cases as those where an alternative linkage pattern of mutations would lead to a change in phenotype [5,27,32-36]. However, these experimental methods of molecular haplotyping are not entirely satisfying because they entail an additional cost and are currently labour-intensive, time-consuming, prone to experimental errors and difficult to automate. Therefore, they do not appear to be good candidates for routine clinical applications and for a generalization at a large scale.

A cheap and relatively straightforward alternative for haplotype reconstruction based on genotype data from unrelated individuals is the use of computational algorithms. The most widely used algorithms developed so far are based either on a parsimony, a maximum-likelihood, or a Bayesian approach (see [37] for a review). In the last decade, numerous investigations based on empirical data and extensive simulation studies have demonstrated that such *in silico *haplotype-inference methods could give effective and accurate prediction of haplotype phases, especially in regions with high LD values between polymorphic sites and small probabilities of recombination events [18,38,39]. Therefore, they could be fairly efficient alternatives to molecular-haplotyping methods when applied to *NAT2 *gene data. Surprisingly, to our knowledge, only three studies have used computational methods to reconstruct *NAT2 *haplotypes and estimate allele frequencies in population samples [40-42]. One explanation for such limited use may be the lack of evidence documenting the performance of *in silico *approaches when applied to actual *NAT2 *data. Indeed, the accuracy of these strategies, compared with molecular methods, needs to be assessed before their applications can be advocated at a large scale. A recent study provided preliminary results on this issue: Xu and colleagues [18] empirically evaluated and compared the accuracy of the Clark's algorithm [43], the expectation-maximisation (EM) algorithm and a Bayesian method implemented in the PHASE program [44] in phase inference at *NAT2*, taken as an example of a locus with pronounced LD over a 850-bp region. In this study, *NAT2 *haplotypes (consisting of five genotyping SNPs at position 282, 341, 481, 590, and 803 nt) were experimentally determined through cloning and sequencing in 81 individuals of European ancestry. They found that all three computational methods provided remarkably accurate and reliable estimates for *NAT2 *haplotype frequencies and individual haplotype phases.

The objective of the present study was to extend this investigation to more precisely assess the performance of the computational approach. We conducted an extensive study based on experimental data from a larger number of samples, issued from populations of various ethnic origin, and tested for haplotypes involving the seven major polymorphic loci of *NAT2*. Furthermore, the larger population samples investigated are of greater significance: as the sample size grows, there is more opportunity to observe rare haplotypes that are the most difficult to infer statistically. This comparative study is designed to evaluate the performance of different haplotyping algorithms and to assess the consistency of their estimates. In addition, it provides information on the impact of various data-set characteristics (sample size, haplotype frequency distribution, haplotype frequencies, deviation from Hardy-Weinberg (HW) equilibrium, ...) on estimation accuracy; we then explore the utility of data-based diagnostics for assessing probable accuracy.

## Results

**Figure 2:**
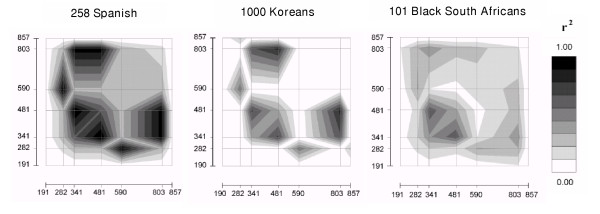
Linkage disequilibrium (r^2 ^value) between SNP markers in the *NAT2 *locus. Graphical representation of the disequilibrium matrices obtained through computation of the r^2 ^coefficient between each pair of markers, for the Spanish, Korean and Black South African samples. The British and Nicaraguan samples provided patterns and levels of LD comparable to those of the Spanish data. For each marker pair, GOLD [60] plotted the color-coded pairwise r^2 ^statistics at the Cartesian coordinates corresponding to marker location, and the plots were completed by interpolation. These graphs point out the strong level of LD between markers at positions 341, 481, and 803, as well as between SNPs located at 282 and 590: these markers are thus strongly predictive of one another. In Black South Africans, LD patterns are less pronounced and more diffuse across marker pairs.

Molecular haplotyping of the *NAT2 *locus revealed between eight and twelve distinct haplotypes in each of the five population samples investigated. The theoretical maximum number of haplotypes for a set of seven biallelic variable sites is 128 (2^7^) if there is random association between polymorphic sites, whereas it is only 8 in the absence of recombination, recurrent and back mutation. Hence, the small number of haplotypes observed at *NAT2 *suggests strong LD over the short physical distance spanning this gene. Indeed, we observed complete or near complete LD for all pairs of SNPs with sufficiently high frequencies (only alleles with frequencies in the range 0.05–0.95 were included in the analysis because estimates of LD for low-frequency alleles in small samples are not informative): 85% of all pairwise r^2 ^values were highly significant (Exact *p*-value <0.0001). Although LD patterns were rather similar among the different population samples, substantial differences in LD levels were observed (Figure 2): the Korean sample, and especially the South African sample, displayed much smaller values of average pairwise r^2 ^(0.27 and 0.20, respectively) than the two European and the Nicaraguan samples (values between 0.39 and 0.57), for which a strong haplotypic structure was observed.

Among the 1608 individuals investigated over the five data sets, 45.5% (732/1608) were either homozygous for all SNP sites or heterozygous at only one SNP site; thus, their haplotype pairs could be assigned directly. Besides, 35.7% (574), 10.0% (160), 0.9% (15), and 7.9% (127) individuals were heterozygous at two, three, four and five SNP sites, respectively. We inferred their haplotype phases with four computational haplotyping methods, and compared the results with those obtained through molecular haplotyping.

Since the Hapar program often provided several equally parsimonious solutions for a given multilocus genotype, it could not resolve a relatively large fraction of heterozygous individuals in each sample and hence, we could not deduce frequency estimates for the haplotypes observed. Therefore, we evaluated Hapar only on its ability to identify the set of haplotypes present in a sample.

### Haplotype identification

Hapar found for each sample the smallest set of haplotypes that could explain the genotype data, and PL-EM, Haplotyper and PHASE provided the list of all the haplotypes selected to appear in at least one of the subjects in the "best" reconstruction, that is when the most likely pair of haplotypes is selected for each individual. The *I*_*H *_indices of the four programs are displayed for each population sample in Table 3. For the British and Korean samples, all computational methods identified exactly the same haplotypes as those determined experimentally. In contrast, in the other three samples, the algorithms sometimes inferred one additional haplotype, that was not actually present, and/or missed one haplotype that was shown to be present by means of molecular haplotyping. Nevertheless, these prediction errors always concerned rare haplotypes of frequency <0.75% (singletons in most cases). The PHASE algorithm performed the best.

**Table 3:** Performance of the four computational methods in haplotype identification, as measured by the *I*_*H *_index.

	Hapar	PL-EM	Haplotyper	PHASE
258 Spanish [45]	0.933 *(1)*	0.933 *(1)*	0.933 *(1)*	0.933 *(1)*
137 Nicaraguans [30]	0.952 *(1)*	0.952 *(1)*	0.909 *(2)*	0.952 *(1)*
112 UK Caucasians [24]	1	1	1	1
101 Black South Africans [24]	0.917 *(2)*	0.917 *(2)*	0.917 *(2)*	1
1000 Koreans [31]	- *	1	1	1

Numbers in brackets indicate the number of haplotypes for which an error of prediction was made. * The size of the Korean sample was too large to be correctly handled by the Hapar program.

### Prediction of individual haplotype phases

We also evaluated and compared the effectiveness of the computational methods in reconstructing haplotype pairs for individuals. Table 4 gives, for each data set and for each algorithm, the individual error rate. Whatever the method, the number of incorrectly reconstructed individuals was remarkably low, with error rates always under 4%. The largest number of mistakes were observed for the African sample and, among the three algorithms tested, PHASE yielded the lowest error rates. Furthermore, it is interesting to note that, in all cases of incorrectly predicted phases, there was no impact on phenotype prediction. Thus, despite these errors, the proportions of slow, intermediate and rapid acetylators in each population sample were in 100% agreement with those deduced from molecular haplotyping.

**Table 4:** Individual error rate in haplotype reconstruction

	PL-EM	Haplotyper	PHASE
258 Spanish [45]	0.39%	0.39%	0.39%
137 Nicaraguans [30]	2.19%	3.65%	2.19%
112 UK Caucasians [24]	0.89%	0%	0%
101 Black South Africans [24]	3.96%	3.96%	2.97%
1000 Koreans [31]	0.30%	0.30%	0.30%

The individual error rate is defined as the ratio of erroneous phase calls to the total number of phase calls (see text).

### Estimation of haplotype frequencies

**Figure 3:**
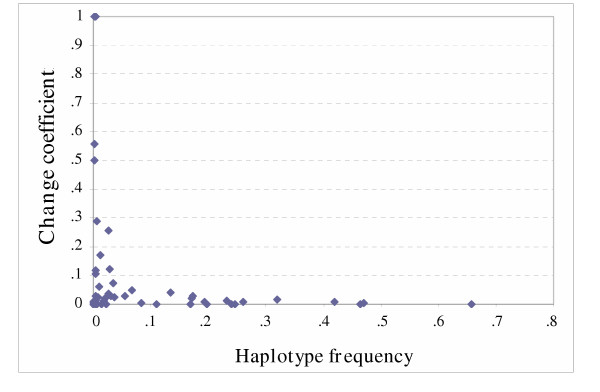
The change coefficient (*C*) as a function of haplotype frequency. The change coefficient reflects the discrepancy between haplotype frequencies deduced from phase-known data and those estimated computationally (here with the PHASE program). All haplotypes occurring in any of the five population samples are considered.

A comparison of the haplotype frequencies determined molecularly with those estimated computationally showed very high concordance. Both PL-EM and PHASE methods provided similarity index (*I*_*F*_) values very close to the maximal value of 1 in all investigated data sets (Table 5). Such high values may be explained by the fact that the *I*_*F *_index gives more weight to common haplotypes whose frequencies are the most accurately estimated by computational algorithms. To investigate the effect of haplotype frequency on estimation accuracy, we plotted the change coefficient (*C*) against the larger of the two haplotype frequencies (Max [, *p*_0*i*_]), for all possible haplotypes with nonzero frequency estimates determined by either analysis in any of the five population samples. As shown in Figure 3, substantial percentage changes >30% occur only at the lowest haplotype frequencies (<0.007). Even the moderate changes (range 10%–30%) occur only when the frequency estimates are <0.03, and any change in percentage value >5% concerns only haplotype frequencies <0.035. Two-thirds (67%) of the haplotype frequency estimates showed either no change or a small change (< 3%). In Table 6, we compared the relative estimation accuracy of PL-EM and PHASE programs by computing the average change coefficient for three classes of haplotype frequencies. The two methods perform similarly for haplotypes with frequencies >0.05, whereas PHASE provides more accurate estimates when rarer haplotypes are concerned.

**Table 5:** Index of similarity (*I*_*F*_) between haplotype frequencies estimated with and without molecular haplotyping information.

	PL-EM	PHASE
258 Spanish [45]	0.996	0.996
137 Nicaraguans [30]	0.986	0.986
112 UK Caucasians [24]	0.994	0.998
101 Black South Africans [24]	0.981	0.988
1000 Koreans [31]	0.997	0.998

Computations of *I*_*F *_indices were based on the haplotype frequency estimates obtained by considering all possible haplotype configurations (with a nonzero probability) inferred for each subject, weighted by their estimated probability. Note that such estimates can be rather different from those obtained by gene counting on the basis of the "best" reconstruction (that is, when only the most probable pair of haplotype is selected for each sampled individual). Since Haplotyper only provides a summary of the frequency with which each haplotype occurred in the "best" reconstruction, it was excluded from the comparison.

**Table 6:** Average change coefficients of PL-EM and PHASE programs computed for three classes of haplotype frequency.

	Haplotype frequency		
	< 1%	1–5%	> 5%
PL-EM	30.6%	8.3%	1.2%
PHASE	17.4%	6.8%	1.2%

### Partially resolved data sets

We also performed similar analyses on six other previously published data sets, in which linkage phase patterns were only partially resolved by molecular haplotyping. These data concerned 844 German [32], 248 Polish [33], 303 Turkish [34], 50 non-caste Dogons from Mali, 52 Gabonese and 60 Caucasians [5]. Haplotype phase information was available for 41%–74% individuals in these six population samples (including phase-resolved genotypes as well as non ambiguous homozygous or simply heterozygous genotypes). The PHASE algorithm was applied on the unphased genotype multilocus data of each of these samples, and a 100% concordance was observed between individual haplotype phase reconstruction through the computational method and the empirically determined linkage patterns, for all investigated data sets (data not shown). This means that, despite the efforts invested, in terms of work, time and money, to resolve mutation linkage phase in a part of each sample, no more information was added by molecular haplotyping than what could be extracted from computational algorithms applied to these data.

## Discussion

This empirical study demonstrates how closely the frequencies computationally estimated from phase-unknown data approximate those from gene-counting estimates based on phase-known data. In the particular case of the *NAT2 *gene, where there is near complete LD between SNPs within the coding region, all *in silico *approaches provided highly effective and accurate estimates for haplotype frequencies and individual haplotype phases. Estimated frequencies of common haplotypes were nearly identical to those empirically determined, whereas rare haplotypes were occasionally miscalled when their presence/absence had to be inferred. As already pointed out by Stephens *et al. *[44] and Lin *et al. *[39] and confirmed in this study, lower-frequency variants are less easily estimated statistically; indeed, there is less contextual information about phase for singletons versus nonsingletons. Thus, for those research questions for which the *NAT2 *common haplotypes are most important, frequency estimates based on the unphased SNP-typing results from unrelated individuals will be sufficient. However, accurate identification of rare haplotypes may be critical for many researchers, such as population geneticists interested in detecting features of recent demographic history that are population-specific or signatures of selective effects in *NAT2 *sequences; as well as for epidemiologists and clinicians concerned with the possibility that rare haplotypes may be important for disease risk or for predicting drug response. In such cases, molecular haplotyping will be necessary to determine linkage phase unambiguously [57].

For a locus such as *NAT2 *where a strong haplotypic structure is observed, all algorithms provided highly effective and accurate results for haplotype reconstruction. Thus, such "ideal" data for statistical inference did not permit to properly discriminate between the different methods investigated. Nevertheless, despite roughly similar performances, slightly better results were observed with the PHASE program. In particular, PHASE outperformed the other programs when frequencies of rare haplotypes have to be inferred. This is consistent with the results of some previous studies which evaluated and compared the performance of several algorithms on both empirical and simulated data [44,54,61]. PHASE provided the most accurate reconstructions, probably because the true haplotypes conformed more closely to the assumptions of the approximate coalescent prior than to those of the Dirichlet prior.

Many factors may influence the estimation accuracy of computational approaches. They can be assessed empirically within a dataset, to be further used as "diagnostics" for predicting potential inaccuracies in estimation caused by features in the relevant data set [38].

Sample size did not appear to have a large effect on the haplotype frequency estimates comparing phase-known and phase-unknown results for the five data sets included in this study. Perhaps the low error rate observed in Koreans is partly due to the huge size of this sample (1000 individuals): an improvement in accuracy of the estimation procedure with increased sample size is indeed expected since information redundancy in the form of multiple copies of the same haplotype in the data set is required for the statistical algorithms to work properly [38,48]. On the other hand, computational methods may also perform best in small samples, in which there is little chance to observe rare haplotypes that are the most difficult to infer statistically. Nevertheless, since the number of new haplotypes is not expected to increase linearly with sample size, the analysis of sufficiently large samples should guarantee a good reliability in the resulting estimates.

Although most of the tested algorithms assume HW equilibrium, significant departures from HW proportions did not seem to have any impact on the accuracy of their predictions. HW equilibrium holds well for the Nicaraguan, UK Caucasian, and Black South African samples (nonsignificant results), whereas the genotype distribution in the Spanish and Korean samples show significant departures from HW proportions (Table 2). The data sets investigated are not the most suitable to evaluate the effect of a deviation from HW equilibrium: significant results for tests of HW ratios are quite close to the threshold value (5%), and in the case of the Korean sample, an excess homozygosity is observed, which should not compromise the algorithm performance. Indeed, in such a case, there is a balance between loss of accuracy caused by violation of HW equilibrium and gain of accuracy caused by the decrease in missing phase information through an excess of homozygotes [38,62].

**Table 2:** The five phase-resolved *NAT2 *molecular data sets investigated.

**Population sample**	**Proportion of phase-unknown multiple heterozygotes^a^**	***NAT2 *gene diversity^b^**	**Deviation from the Hardy-Weinberg equilibrium (exact p-value)^c^**
258 Spanish [45]	66.7%	0.65	**0.012**
137 Nicaraguans [30]	59.1%	0.70	0.072
112 UK Caucasians [24]	52.7%	0.69	0.222
101 Black South Africans [24]	63.4%	0.86	0.122
1000 Koreans [31]	50.0%	0.52	**0.016**

All population samples were genotyped for the same seven nucleotide changes (G191A, C282T, T341C, C481T, G590A, A803G, G857A), except Koreans where the C190T mutation was investigated instead of G191A.

^a^Proportion of multiply heterozygous individuals with ambiguous genotype whose phase has been resolved molecularly.

^b^Expected heterozygosities for the *NAT2 *haplotyped system were estimated as  where n is the number of gene copies in the sample, and pi is the sample frequency of the i-th haplotype.

^c^The significance of deviations from Hardy-Weinberg equilibrium was tested for genotypic data with known gametic phase using the random-permutation procedure implemented in the Arlequin package [51]: a Fisher's exact test using a Markov chain random walk algorithm was performed for each data set. The resulting p-values were considered significant if inferior to 0.05 (significant p-values are shown in bold).

Among the five data sets investigated in this study, Black South Africans displayed the highest error rate in haplotype computational inference. One possible explanation may be the presence in this sample of a large number of different multiple heterozygotes with ambiguous multilocus genotypes, occurring at roughly similar frequencies (this is reflected in the high *NAT2 *gene diversity displayed by this population sample (Table 2)). Indeed, both the number of different ambiguous multiple heterozygous genotypes and their relative frequencies have been shown to be of high importance in the assessment of haplotype estimation accuracy [57]: both would be good indicators of the difficulty level of a given data set for haplotyping algorithms. The existence of many different multilocus genotypes uniformly distributed implies that many different haplotypes occur at low frequency, and that, consequently, greatest error and uncertainty occur in the estimation of haplotype frequencies (since no single haplotype is overwelmingly frequent). In contrast, the presence of a small number of multiple heterozygous genotypes at proportionately high frequencies implies that some individual haplotypes exist at high frequencies, and the estimation of those haplotype frequencies will be easier and accomplished with greater accuracy [38,57]. In such cases, molecular haplotyping may add little information for the resolution of haplotype phases.

The amount of LD between SNP markers may be another determining factor for the prediction of estimation reliability since when multiple polymorphic sites display little disequilibrium, as was observed in the African sample compared to the others, a large proportion of the chromosomes may occur as uncommon or rare haplotypes, implying a greater difficulty level in the statistical inference of haplotypes.

Therefore, we advocate to examine beforehand the unphased *NAT2 *genotype data for both the frequency distribution of multiply heterozygous genotypes and the level of LD between polymorphic markers; this will allow to assess the difficulty level displayed by the data set for statistical inference, and hence, to predict the ability and accuracy with which computational algorithms would infer haplotype phases from such data.

Of course, statistical methods can be used in conjunction with experimental methods to provide more accurate estimates of individual haplotypes. It has been claimed that the ability of certain computational methods to accurately assess the uncertainty associated with each phase call gives them the substantial practical advantage of allowing experimental effort to be directed at sites and/or individuals whose phases are most difficult to reconstruct statistically or that are critical to the conclusions of the study [20,44,61]. However, in our study, we observed that most erroneous phase calls inferred at the individual level were strongly supported, with a probability close to the maximal value of 1. Thus, these errors could not have been avoided since they would not have been selected for the molecular targeting. This stresses why, in the case of the discovery of a novel *NAT2 *allele through computational haplotyping methods, the unusual linkage pattern should always be confirmed by cloning and sequencing the allele under question, as advocated by Cascorbi and Roots [25] for novel allelic combinations detected by molecular techniques.

Throughout this study, we assumed that the *NAT2 *linkage patterns molecularly determined were the "true" ones, and hence, that there was no error in the haplotype assigments based on experimental methods. However, molecular techniques may have experimental error rates as high as the rate of statistical error associated with the computational haplotype determination algorithms [19]. Indeed, molecular haplotyping bears the risk of false positive or false negative allele-specific amplification (because of the nucleotide-dependent specificity of that technique) as well as uncomplete or non-specific digestions with the enzymes used in restriction analyses [25]. In the present study, we have estimated the computational error rates to be of no more than 3–4% for all investigated algorithms. This is not higher than the corresponding error rate from molecular haplotyping techniques, on the order of 2–3% [20]. Therefore, it is difficult to determine whether the discrepancies observed between experimental and computational estimates are actually due to statistical errors from algorithms; they may be due to technical errors during manipulations and the molecular data used as a reference for comparisons might be wrong.

The disadvantage of *in silico *approaches is that algorithmic techniques are statistical and require the analysis of a population rather than a single individual. This is not a limitation in clinical trials and epidemiological surveys, which are always performed on a cohort basis. In clinical pharmacy, however, if a specific individual's haplotypes are of interest to predict his response to drug treatment, his unphased multilocus genotype must be combined with a standard reference set of haplotypes to infer the phasing [20]. This implies a thorough knowledge of the *NAT2 *genotypic distribution in the ethnic population from which this individual was drawn.

## Conclusion

This study demonstrates that computational methods can provide an effective and accurate prediction of haplotype phases, in the particular case of the *NAT2 *gene which displays high values of LD between polymorphic sites. The objective of this study is not to advocate the systematic use of computational approaches for *NAT2 *haplotype inference at the expense of molecular haplotyping methods. We are convinced that these last ones remain the most reliable and effective way to resolve linkage phase patterns and that they can produce, for a fixed sample size, much more precise estimates of haplotype frequencies than other approaches [63]. However, the considerable effort required to obtain and analyse individual chromosomes make alternative designs preferable; and the *in silico *approach appears to be the most practical one. Thus, for researchers not willing to invest time and money in the preliminary step of *NAT2 *haplotype reconstruction, the use of computational algorithms constitutes a safe and effective way to get reliable haplotypic data on which further analyses could be carried on. Once haplotypes are constructed, various statistical methods can be applied on *NAT2 *haplotype data to detect allele-disease associations or to classify patients according to their acetylation status.

## Methods

### NAT2 molecular data sets

To evaluate the performance of *in silico *approaches in *NAT2 *haplotype reconstruction, we based our study on data collected from the literature, for which linkage phase was resolved directly through molecular haplotyping. Molecular data from five previously published data sets were analysed: they concerned 258 Spanish from Central Spain [45], 137 Nicaraguans with a Central American Indian-European mixed origin [30], 112 British from the Cambridge area [24], 101 Black South Africans (mostly Tswana-speaking people) [24], and 1000 Koreans [31]. All subjects included in these studies were randomly selected, unrelated healthy volunteers whose ethnic origin had been clearly defined. In each population sample, seven SNPs were typed at *NAT2 *for all individuals (no missing data), and mutation linkage phase of all multiply heterozygous individuals was resolved molecularly through allele-specific PCR and restriction mapping. A summary description of the data sets is given in Table 2. These data provide an opportunity to compare haplotype frequencies estimated by direct gene counting on experimentally haplotyped data with haplotype frequencies estimated by haplotyping algorithms when phase information is ignored.

Throughout this report, we will use the term «phase-known» to refer to the individual's genetic constitution for the *NAT2 *haplotyped system, including the linkage phase of the component SNP alleles. Whereas we will use the term "phase-unknown" to refer to an individual's multilocus genotype in the absence of phase information.

### Computational haplotyping methods

We evaluated the ability of four population-based haplotype inference methods to reconstruct *NAT2 *haplotypes from the phase-unknown genotype data.

#### Hapar

The first method is based on maximum parsimony: it searches for a set of minimum number of haplotypes that explain the observed genotype samples. Clark's method, the first developed algorithm for haplotype reconstruction [43] which can be viewed as a sort of parsimony approach, requires homozygote or single-site heterozygote in the sample to start its inferential cascade. Wang and Xu [46] overcame this limitation by designing an algorithm with a global optimization goal. This method, recently implemented in the Hapar program [46], was tested at its default settings on the phase-unknown *NAT2 *data.

#### PL-EM

We also applied the EM algorithm [47] to obtain the maximum-likelihood estimates of haplotype frequencies in the samples, given the observed data [48-50]. This algorithm starts with initial arbitrary values of haplotype frequencies and iteratively updates the frequency estimates, to maximize the log-likelihood function, until convergence is reached. Several EM-based algorithms have been developed. We used three different implementations, Arlequin [51], HAPLO [49] and PL-EM [52], that all gave us identical results on comparable analyses (data not shown). Thus, we presented only the results obtained with the PL-EM program: this software implements an algorithm derived from the standard EM but incorporating the computational strategy of partition-ligation [53] to handle a larger number of loci. We performed 50 independent runs with different initial conditions to minimize chances of local convergence so as to ensure finding the global maximum likelihood estimates. For a given unphased genotype pattern, the probability of each possible haplotype configuration was calculated by using the estimated population haplotype frequencies, and all compatible haplotype phases with nontrivial probabilities were generated. The haplotype pair with the greatest probability was considered to be the haplotype phase for each individual, and population haplotype frequencies were estimated as a function of each inferred haplotype pair, weighted by their estimated probability.

#### Haplotyper and PHASE

Finally, two Bayesian statistical methods based on Gibbs sampling procedure were applied to the phase-unknown *NAT2 *data. Such methods treat the unknown haplotypes as random quantities and combine *prior information *-beliefs about what sorts of patterns of haplotypes are expected to be observed in population samples- with *the likelihood *-the information in the observed data [54]. The conceptual difference between the two investigated Bayesian algorithms lies in the prior information incorporated into the statistical model. The algorithm implemented in the Haplotyper program [53] uses a Dirichlet prior distribution, which assumes that the genetic sequence of a mutant offspring does not depend on the progenitor sequence [54]. Instead, the algorithm implemented in the PHASE program [44] uses a prior approximating the coalescent, which is one of the evolutionary models most commonly used in population genetics (see [55] for a review): it assumes that unresolved haplotypes will tend to be the same as, or similar to, known haplotypes. We employed the lastest version of PHASE (PHASE v 2.1 [54]) to evaluate the performance of this method, using the default parameter values in the Markov chain Monte Carlo simulations. For each data set investigated, we applied the algorithm ten times with different seeds for the random number generator, and checked for consistency of the results across the independent runs in order to verify that the algorithm did not converge to a local, rather than global, mode of the posterior distribution. We chose the results from the run displaying the best average goodness-of-fit of the estimated haplotypes to the underlying coalescent model. Besides, to evaluate the Bayesian algorithm implemented in Haplotyper, we performed 20 independent runs of the program on each sample. This software could not be run directly on the 1000-Korean sample as it can only handle 500 individuals at most per data set. To circumvent this limitation, we randomly generated ten pairs of complementary data sets, each composed of 500 individuals, and we ran Haplotyper on each of them. Results were averaged over the ten complete data sets. Both programs Haplotyper and PHASE provide a list of the most likely pairs of haplotypes for each subject. They also quantify the uncertainty associated with each phase call by outputting an estimate of the probability that each call is correct. This prevents inappropriate overconfidence in statistically reconstructed haplotypes.

### Measures of estimation accuracy

Computational algorithms of haplotype reconstruction may be used for many different purposes. We focus here on three particular tasks: finding the list of all haplotypes present in a sample, inferring the most likely pair of haplotypes for each sampled individual, and estimating haplotype frequencies in the population. Thus, three different measures of accuracy were used to evaluate the performance of the tested algorithms.

#### -haplotype identification

To assess accuracy in terms of haplotype identification, we used the *I*_*H *_index introduced by Excoffier and Slatkin [48]. It compares the number of different haplotypes detected experimentally with the number of different haplotypes inferred by the computer programs. We considered that a given haplotype is identified as being present in the true sample if its estimated frequency is above the threshold value of 1/(2n) in a population sample of n individuals. *I*_*H *_is given by:



where *k*_*true *_is the number of haplotypes in the true sample, *k*_*est *_is the number of estimated haplotypes with frequency above the threshold, and *k*_*missed *_is the number of true haplotypes not identified in the sample.

Values of *I*_*H *_can vary between 1 (when the computational identified haplotypes are exactly the same as those determined experimentally) to 0 (when none of the true haplotypes are identified computationally).

#### - reconstruction of the haplotypes of each sampled individual

We specified the haplotype pair for an individual by choosing the most probable haplotype pair consistent with the individual's multilocus genotype. We measured performance by the *individual error rate*, which is the proportion of individuals whose haplotype pairs were incorrectly inferred by the program [53].

#### - estimation of sample haplotype frequencies

To examine how close the computationally estimated haplotype frequencies are to the observed frequencies in the phase-known data, we used the similarity index *I*_*F *_of Renkonen [56], defined as the proportion of haplotype frequencies in common between the estimated and observed frequency distributions [18,48].



where  and *p*_0*i *_denote, respectively, the estimated and observed sample frequency of the i-th haplotype. This measure incorporates all *h *haplotype frequencies and thus captures the overall difference between estimated and observed frequencies for a particular data set. It varies between zero, when true haplotypes have estimated frequencies tending to zero, and one, when observed and estimated frequencies are identical.

Since this index gives more weight to the high-frequency haplotypes, we used a second criterion to assess the accuracy of computational algorithms in haplotype frequency estimation: the change coefficient *C*, defined in Tishkoff *et al. *[57] as



where Max [, *p*_0*i*_] indicates the maximum value of  or *p*_0*i*_.

This coefficient measures the percentage change in haplotype frequencies across the two information conditions (phase-known versus phase-unknown data). *C *coefficients were computed for each possible haplotype in each population. The value of *C *ranges from 0 to 1, with 0 indicating that the estimated and observed frequency are identical. The maximal value of 1 indicates that molecular haplotyping showed either the presence of a haplotype that was assigned a zero through computational haplotyping, or *vice versa*.

### Measure of pairwise LD between SNP markers

We used the phase-known data to quantify the amount of LD between all pairs of polymorphic sites by computing the correlation coefficient r^2 ^[58] for each population sample separately. These statistics are expected to be 1 (perfect LD) when the variation is segregating in a population as only two distinct haplotypes. Statistical significance of LD between pairs of sites was assessed by Fisher's exact tests. Computations were performed with the software PowerMarker v3.21 [59], and a graphical summary of disequilibrium matrices was displayed by the GOLD program [60].

## Abbreviations

Single nucleotide polymorphism (SNP)

Expectation-Maximisation (EM) algorithm

Linkage disequilibrium (LD)

N-acetyltransferase 2 (NAT2)

## Authors' contributions

AS participated in the conception and design of the study, in collection of data, in performing all the computational analyses, and in drafting the manuscript. PD participated in the design of the study, in interpretation of data and in revising the article critically for important intellectual content. All authors read and approved the final manuscript.
